# Flexible Superhydrophobic and Superoleophilic MoS_2_ Sponge for Highly Efficient Oil-Water Separation

**DOI:** 10.1038/srep27207

**Published:** 2016-06-02

**Authors:** Xiaojia Gao, Xiufeng Wang, Xiaoping Ouyang, Cuie Wen

**Affiliations:** 1 School of Materials Science and Engineering, Xiangtan University, Hunan 411105, China; 2 School of Aerospace, Mechanical and Manufacturing Engineering, RMIT University, Bundoora, Victoria 3083, Australia

## Abstract

Removal of oils and organic solvents from water is an important global challenge for energy conservation and environmental protection. Advanced sorbent materials with excellent sorption capacity need to be developed. Here we report on a superhydrophobic and superoleophilic MoS_2_ nanosheet sponge (SMS) for highly efficient separation and absorption of oils or organic solvents from water. This novel sponge exhibits excellent absorption performance through a combination of superhydrophobicity, high porosity, robust stability in harsh conditions (including flame retardance and inertness to corrosive and different temperature environments) and excellent mechanical properties. The dip-coating strategy proposed for the fabrication of the SMS, which does not require a complicated process or sophisticated equipment, is very straightforward and easy to scale up. This finding shows promise for water remediation and oil recovery.

Oil spillage and the organic solvents discharged by chemical industries are primary pollutants of water resources, and have resulted in significant energy losses, serious environmental pollution and consequent ecological problems. Under such circumstances, superhydrophobic porous materials, such as sponges, meshes, fabrics, and membranes, have stimulated great interest because of their capacity for selective absorption/separation of oils or organic solvents while repelling water completely^1^,^2^,^3^,^4^. Among these potential materials, superhydrophobic sponges have many attractive inherent advantages such as low price, low density, excellent flexibility and high mechanical stability, and they can be flexibly utilized to selectively absorb oil and solvents. A number of strategies have been designed to render sponges the superhydrophobicity, but these strategies require complicated and repetitive treatment processes such as oxygen plasma pre-treatment, hydrothermal treatment and vapor phase deposition^5^,^6^,^7^,^8^. Therefore, besides important properties in terms of high absorption capacity and selectivity, strong chemical inertness, and environmental friendliness, a novel superhydrophobic sponge with a simple and effective fabrication process still needs to be explored.

As a noteworthy layered semiconductor in the transition metal dichalcogenide material family, molybdenum disulfide (MoS_2_) has attracted much attention recently owing to its excellent physical properties such as high carrier mobility, photoconductivity, environmental sensitivity, and mechanical properties^9^,^10^,^11^. These properties qualify MoS_2_ sheets as promising for applications in catalysis, transistors, batteries, photo detectors, and flexible electronic devices^12^,^13^,^14^,^15^,^16^. Besides their outstanding physical and chemical properties, MoS_2_ materials have recently been reported to possess unusual wetting behavior, similar to that of graphene, and its hydrophobicity is thickness independent, *i.e*. a perfect MoS_2_ monolayer (without airborne contaminants) on SiO_2_/Si substrates is intrinsically hydrophilic with a water contact angle (WCA) of 70°, while multiple layer films (beyond three layers) are slightly hydrophobic. Interestingly, when the hydrocarbons and water present in air adsorb onto the clean film, aged MoS_2_ sheets display a stabilized hydrophobicity^17^,^18^. For other two-dimensional materials such as graphene films fabricated by chemically or thermally reduced methods, their hydrophobicity can be easily increased to superhydrophobicity and superoleophilicity using the surface roughness effect in conjunction with surface chemistry modification via ultrasonication in acetone/water^8^,^19^. However, to the best of our knowledge, no studies involving MoS_2_-basedmaterials with both superhydrophobic and superoleophilic properties have been reported to date. Basing on these properties, the materials could be employed for the separation and absorption of oil and organic contaminants from water.

In this paper, we first report a simple and inexpensive dip-coating method for the fabrication of superhydrophobic and superoleophilic MoS_2_ sponges (SMSs). The SMSs absorb a broad variety of oils and organic solvents with high selectivity and absorption capacities, excellent chemical inertness, good recyclability, and outstanding mechanical properties. Moreover, this modified sponge could be used in conjunction with a vacuum apparatus for the continuous absorption and removal of oil pollutants from water surfaces.

## Results and Discussion

### Preparation and characterization of MoS_2_ nanosheet sponge

**Figure 1:**
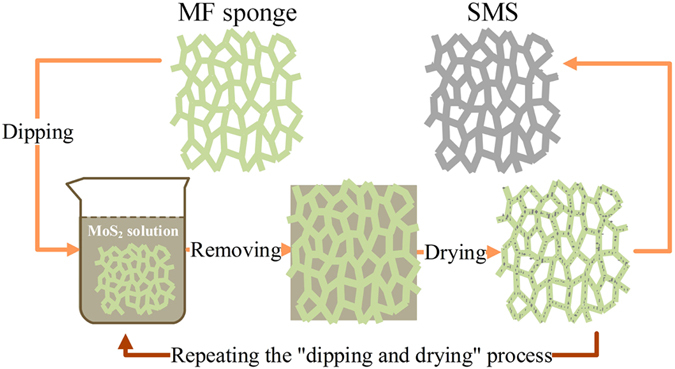
Schematic illustration of the fabrication process for a superhydrophobic and superoleophilic MoS_2_ sponge for oil-water separation.

The preparation procedure of the SMSs is illustrated in Fig. 1, and the main steps are as follows. Firstly, the MoS_2_ nanosheets utilized were obtained by exfoliation of economical powdered MoS_2_ crystals using ultrasonication in ethanol solvent. Secondly, a commercial melamine-formaldehyde sponge (denoted MF sponge), which is a common three-dimensional (3D) porous material with the ability to absorb both water and oils or organic solvents, was used as a frame for the MoS_2_ coating. MF sponge was immersed in the MoS_2_ nanosheet solution with ethanol by a squeezing and vacuum degassing procedure, and MoS_2_ nanosheets were assembled on the MF sponge into a 3D MoS_2_ sponge with a color change from white to black. Finally, the coated sponge was directly dried to yield the SMS. This “dipping and drying” process above was repeated at least three times, ensuring a uniform, consistent and continuous MoS_2_ nanosheet coating. No MoS_2_ fragments detached from the sponges after manual squeezing, indicating that the MoS_2_ nanosheets were physically coated onto the sponge skeletons with significant adhesion, which is attributed to the mechanical flexibility of the MoS_2_ nanosheets and strong van der Waals interactions between the sponge and MoS_2_ nanosheets^9^,^12^,^20^. This proposed process for the fabrication of SMSs is simple and convenient, and does not require the use of any costly organic solvents or a complicated treatment.

Optical microscopy illustrates that MoS_2_ nanosheets extracted from MoS_2_ crystals have a large individual planar structure with several micrometers in width (Fig. S1). Transmission electron microscopy (TEM) further reveals that the thickness of the sheets ranges from dozens of nanometers to several hundred nanometers, this originates from the restacking of single layer MoS_2_ sheets. Selected area electron diffraction analysis reveals hexagonal spots in selected regions of the large sheets (Fig. S2). The individual MoS_2_ nanosheets consist of a number of rough surfaces and folded edges, with a micro/nano-textured structure, which is fundamentally important to the wettability of a surface. To demonstrate the hydrophobic property of the MoS_2_ nanosheets, water contact angle (WCA) measurement was performed on the surface of MoS_2_ films which were deposited on an aluminum substrate using a dip-coating method. It was observed that the MoS_2_ films are strongly hydrophobic with a WCA of 122° ± 3° (Fig. S3).

**Figure 2:**
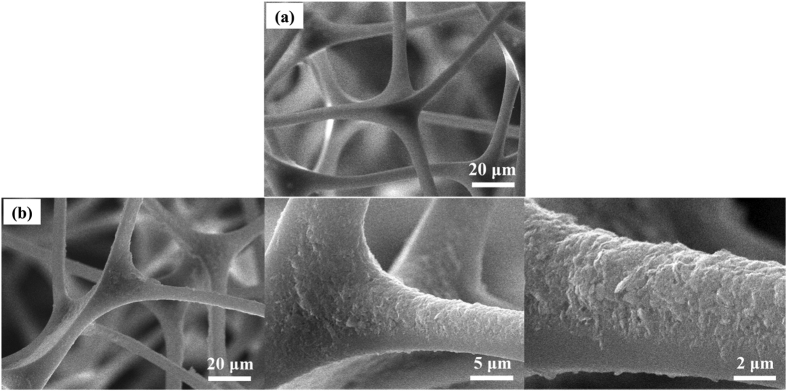
(**a**) Typical SEM images of the purepolymer sponge; (**b**) Typical SEM images of the SMS (higher magnification SEM images are illustrated in order from left to right).

Scanning electron microscopoy (SEM) was used to examine the morphological evolution of the sponge before and after the hydrophobic modification. As shown in Fig. 2, the sponge before and after coating with the MoS_2_ nanosheets display exactly the same porous structure, which is an inherent 3D interconnected porous structure with macro pores of hundreds of micrometers, thus confirming that the small modification does not damage the original structure of the sponge or block the pores inside it. These characteristics are beneficial for the rapid uptake of oil, as the open-pore network permits the rapid transport of gas and liquid in the sponge. It is clear that the smooth skeletons of the original sponge are covered with MoS_2_ nanosheets after dip-coating (Fig. 2b). A higher magnification SEM image of the MoS_2_-coated sponge reveals hierarchical structures that exist in the form of crater-like protrusion, which are the stacked MoS_2_ nanosheets with micro/nano-scale folded edges. Like the surface structure of a lotus leaf, these hydrophobic MoS_2_ nanosheets in combination with the micro-porous structure of the sponge create a doubly roughened surface, which leads to a composite interface in which air has become trapped within the grooves beneath the liquid, therefore achieving superhydrophobicity (the so-called Cassie-Baxter model)^21^.

### Wettability behavior

**Figure 3:**
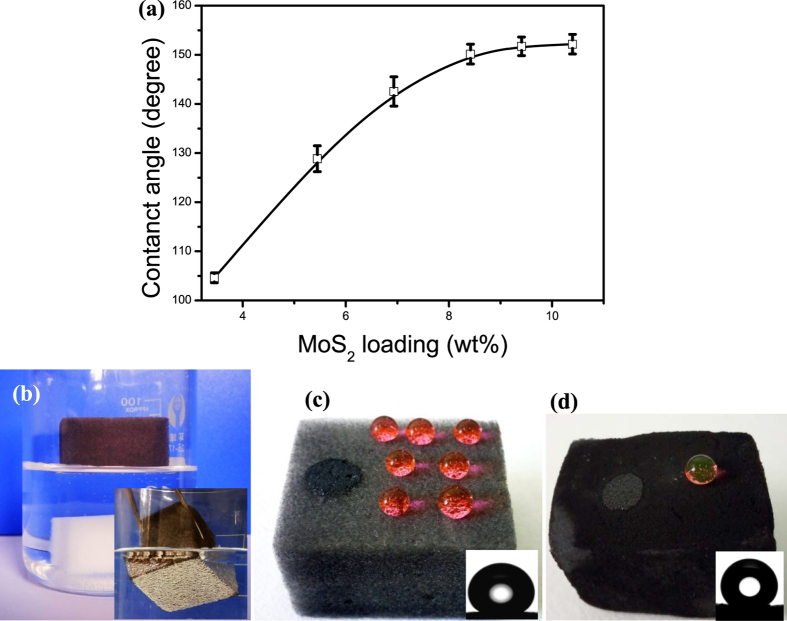
(**a**) Effect of MoS_2_ nanosheet loading on the WCA of the pure sponge; (**b**) Photograph of SMS after being placed into water - the inset is a photograph of the SMS partially immersed in water by force; (**c**) Water droplets (in red) -as quasi-spheres and gasoline trace (marked by a circle) on the surface of the SMS - Inset: optical image of a water droplet on the prepared sponge; (**d**) Photograph of water droplets as quasi-spheres and gasoline trace on the surface of the MoS_2_ sponge after oil-saturated burning - Inset: optical image of a water droplet on the burned sponge.

The raw MF sponge exhibits a typical superhydrophibic and superoleophilic behavior (Fig. S4). To control the different loadings of the MoS_2_ nanosheets on the superhydrophobic property of SMS in the repeated “dipping and drying” process, we defined the weight ratio *W*_MoS2_/*W*_sponge_ (*W*_sponge_ and *W*_MoS2_ are determined by initial weight and weight of MoS_2_-coated sponge, which are weighed immediately after being taken out of the oven to avoid moisture absorption, respectively) as the loading index. Figure 3a shows the variation in the MoS_2_ loading index with its corresponding WCA values. It can be seen that the WCA increases rapidly when increasing the MoS_2_ loading at low concentrations. As the MoS_2_ loading index increases to 8.4% or greater, water droplets attained quasi-spherical shapes on the sponge surfaces with CAs of 150° ± 2°, indicating superhydrophobic behavior. These results suggest that an unsaturated coating degrades the superhydrophobicity of the coated sponges, while an oversaturated coating brings no further improvement in superhydrophobicity and may block the sponge pores. Thus superhydrophobic sponges with a 9.4% MoS_2_ loading were employed in the following study.

As shown in Fig. 3b, a fabricated SMS (black color) floated on a water surface and no water uptake was found, while a pure MF sponge (white color) sank to the bottom of the beaker. When the SMS was immersed in water under an external force (inset of Fig. 3b), the surface of the superhydrophobic sponge appeared like that of a silver mirror, suggesting that this sponge features Cassie-Baxter surfaces. This is due to a composite interface in which a uniform air layer has become trapped between the water and sponge surfaces. Figure 3c shows the water droplets attained near-spherical shapes and rolled off with ease when placed on the surface of the SMS. The inset in Fig. 3c is an optical image of a water droplet (10 μl) on the surface of the SMS with a WCA of 151° ± 2°. By contrast, when gasoline was dropped onto the surface of the SMS, it was immediately absorbed by the sponge, as indicated by the circled area in Fig. 3c, demonstrating its superoleophilic property. The superhydrophobic and superoleophilic surfaces demonstrated here can be attributed to the combination of the micro-porous structure of the sponge, the hydrophobic chemical property of the MoS_2_ nanosheets, and the micro/nano-textured structure of the MoS_2_ nanosheets on the sponge skeletons.

**Figure 4:**
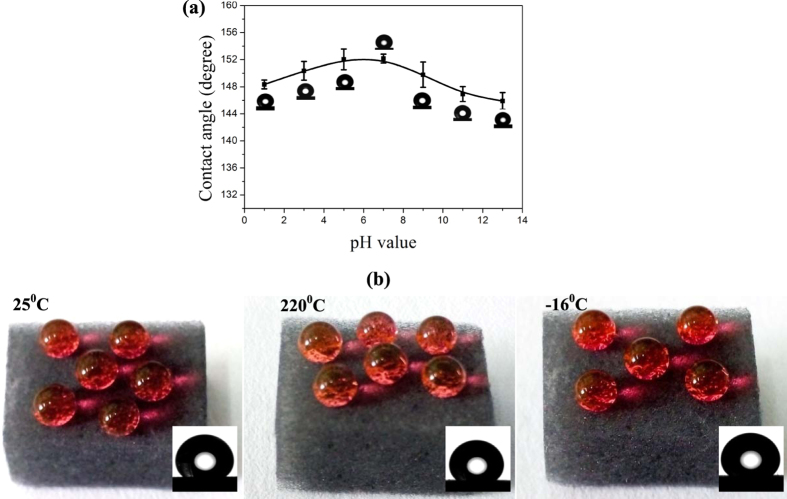
(**a**) Relationships between pH values and WCAs of the SMS after 24 h immersion in aqueous solutions with different pH values; (**b**) Photograph of water droplets on the surface of MoS_2_ sponge after exposure in room (left), hot (center), and cold (right) environments, respectively - inset: optical image of a water droplet on the exposed SMS.

To further evaluate chemical inertness, the hydrophobic stability of the SMS over different pH values and temperatures was tested. The SMS was immersed in aqueous solutions with a broad pH range (1–13) for 24 h and tested. As shown in Fig. 4a, the WCAs of the sponges immersed in aqueous solutions with different pH values are still greater than 145° ± 2°, suggesting that their strong hydrophobicity is resistant to corrosive environments. As shown in Fig. 4b, the WCAs of SMSs exposed to hot (220 °C) and cold (−16 °C) environments for 1 h are the same as in a room (25 °C) environment, which indicates that their wettability is also highly independent of temperature. These features show the superhydrophobic MoS_2_ sponge is highly stable and robust in various harsh environments, which may further extend its potential use.

#### **Oil/organic solvents - water separation**

**Figure 5:**
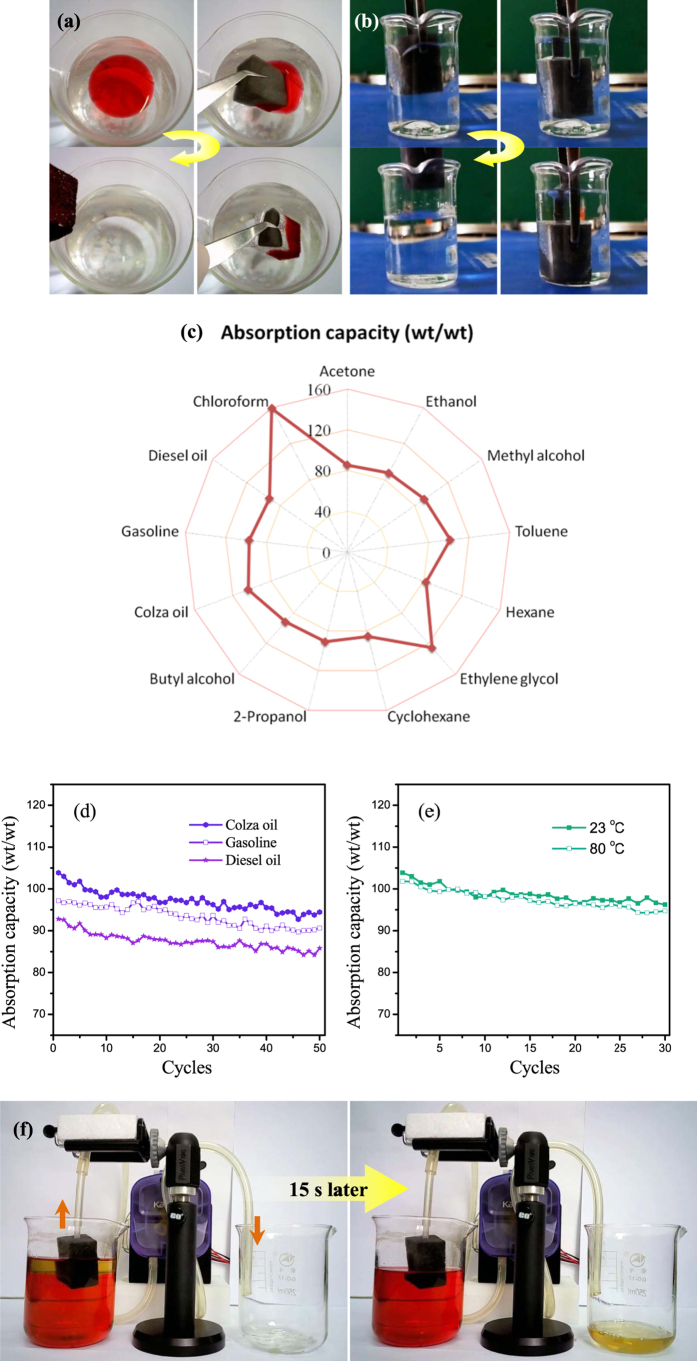
Photographs of: the adsorption process of (**a**) rapeseed oil (dyed with Sudan III) and (**b**) chloroform; the adsorption process of water using MoS_2_ sponge; (**c**) the absorption capacities of the MoS_2_ sponge toward oils and organic solvents; (**d**) the absorption recyclability of the MoS_2_ sponge with different kinds of oils; (**e**) The absorption recyclability of the MoS_2_ sponge at different temperatures; (**f **) continuous collection of gasoline *in situ* from a water surface with the apparatus.

Due to its high porosity, superhydrophobicity and robust stability, the SMS is an excellent candidate for the clean-up of oils and organic solvents in water. Here we chose two organic solvents with different densities, rapeseed oil and chloroform, as model absorbates to verify how SMS would respond as an effective absorbent for organic contaminants. As shown in Fig. 5a, once a piece of SMS was dropped into contact with a layer of the rapeseed oil (dyed with Sudan III) on a water surface, the SMS completely absorbed the oil, resulting in a transparent region of clean water which was originally contaminated by the oil (see detail in Movie S1).The process finished within a few seconds, suggesting a useful route for cleaning up oil spillages. Similarly, chloroform, which sinks to the bottom of water, was also rapidly sucked up by the SMS (Fig. 5b, see detail in Movie S2). Such fast absorption kinetics of the SMS is attributed to the combination of its high porosity, capillary action, and oleophilic nature.

As shown in Fig. 5c, the SMS exhibits excellent absorption capacities towards a wide range of oils (rapeseed oil, gasoline, and diesel oil) and organic solvents (Acetone, Ethanol, Methyl alcohol, Toluene, Hexane, Ethylene glycol, chloroform, Cyclohexane, 2-Propanol, and Butyl alcohol), and absorbs up to 82–159 times its own weight, depending on the density of the absorbates. In particular, the SMS shows an absorption capacity of 93 wt/wt for diesel oil and 159 wt/wt for chloroform, respectively. These absorption capacities are significantly higher than those of commercial PP fabrics and many previously reported high-performance absorbent, *e.g*. ~20 times for nanowire membranes, ~33 times for micro-porous polymers, and 15–25 times for the CNT/PDMS-coated PU sponge, and are comparable to those of ultralight carbon aerogels or sponges for similar oils and solvents^22^,^23^,^24^,^25^. It should be noted that the fabrication process for our SMS is simple and easy to scale up.

To further test the recyclability of SMS for the clean-up of oil, we used typical oils (rapeseed oil, gasoline and diesel oil) as model absorbates to investigate the cyclic absorption/squeezing behavior of the sponge. After absorbing all the oil, the sponges could be squeezed out mechanically to harvest the absorbed oils. Figure 5d shows the recyclable use of the SMS for the absorption of different oils. It is evident that the recyclable absorption behavior of the SMS for three kinds of oils is analogous, *i.e*. a slight deterioration in absorbency was observed over 50 repetitions, indicating its good recyclability. This decrease is due to the residual oil inside the sponges which could not be removed by manual squeezing during each cycle. This recyclable absorption behavior is obviously stronger than those of previously reported sponges, *e.g*., the cycles of nanoparticle copper coated sponges is less than 20, which decreases rapidly with increasing cycle^26^, the absorption capacities of the graphite-based sponge deteriorates rapidly after two cycles^8^. Importantly, the water-repelling behavior of the sponges just decreased slightly after 20 cycles of the absorption/squeezing test, as evidenced by a high WCA of 145° ± 2° (Fig. S5). As a consequence of its high chemical inertness, the superhydrophobic sponge is still robust in high temperature environments (Fig. 5e), *e.g*. its oil absorption capacity after 30 cycles of the absorption/squeezing test in 80 °C is almost as same as in room temperature.

For practical and commercial applications, it is essential to develop a novel, continuous, and convenient collection method^22^,^27^. Here, we achieved continuous collection of oil *in situ* from a water surface based on a simple combination of SMS with pipes and a peristaltic pump. As demonstrated in Fig. 5f, only the pure oil was absorbed by the SMS and flowed along the pipes to the collecting cup, leaving the SMS continuously able to collect the oil (see detail in Movie S3). We also investigated the oil-collection performance of the pump on a water surface with simulated waves, the results show that the shaking of the SMS on the water surface did not affect the oil-separation efficiency because of its buoyancy (Fig. S6 and Movie S4). Moreover, the collection of diesel oil via this pumping apparatus can be maintained for more than 10 hrs without an obvious decrease in flux, indicating the long-time working stability of the SMS (Fig. S7 and Movie S5). This novel oil-collection technique makes the separation of oil-water emulsions easier and faster, which brings SMS a step closer to practical application in oil-water separation.

### Flame-retardance and burning for regeneration

Most organic solvents and oils require separation are highly flammable when ignited, and so it is beneficial to further study the flame-retardant property of the SMS^24^. Here, the combustion behavior of the SMS and MF sponge was investigated using burning tests. It is clear that the MF sponge utilized in this work has a flame-retardant property, and the sponge after superhydrophobic modification inherits this advantage (Figs S8a,b), which indicate sits potential for reducing the risk of fire and explosion. Gasoline was used as a model oil to investigate the combustion behavior of the oil-saturated SMS. The result shows that the gasoline absorbed by the SMS extinguishes less than 70 seconds after being ignited, leaving behind a half-burned sponge (see Fig. S8c). The total weight of the residue is 56% of the original weight, confirming the flame-retardant property of the SMS. More importantly, the SMS can be reused several times by directly burning it in air, as is done with other porous polymer and BN sorbents^24^,^28^. As shown in Fig. 3d, the residue from a burned SMS exhibits dark color, partial shrinkage in volume, and a slightly reduce water contact angle (143° ± 3° after the first cycle), which attributes to the presence of carbonaceous matter. Oil can be taken up again at least five times with a slight decrease in capacity (Fig. S9). This is in contrast to other carbon-based materials and polymer-based sorbents that have higher initial capacities, but cannot withstand such harsh conditions and therefore can only be used several times before their porosity is completely filled by carbonaceous matter^7^.

### Mechanical stability

Its excellent mechanical properties are also of great importance for SMS in order to realize its applications in oil-water separation. Here, compression experiments were performed to evaluate the mechanical performances of SMS. The prepared SMS completely recovers its original shape without plastic deformation after compression, and this is maintained without apparent structural damage even after 1000 cycles of a 50% compression test (Fig. S10), indicating excellent flexibility and mechanical robustness. Importantly, the SMS can be not only highly compressed but also bent and twisted, as shown in Fig. S11. After releasing the loading, it rapidly recovers its original shape without structural fatigue. These excellent mechanical properties of SMS are attributed to its unique structural design, which partly transfers a load from the MoS_2_ nanosheets to the polymer skeletons under mechanical deformation. Once the load is removed, the polymer skeletons return to their original configurations, allowing the SMS to recover its initial shape. Interestingly, the SMS still exhibits robust mechanical stability in burning conditions, *e.g*. after one cycle of burning it can be compressed easily, and completely recovers its original shape without mechanical failure (Fig. S12).

## Conclusions

In summary, we have reported on a superhydrophobic and superoleophilic MoS_2_ sponge for highly efficient separation and absorption of oils and organic solvents from water. This novel sponge exhibits excellent absorption performance (including good selectivity, high capacity, and good recyclability), extraordinarily robust stability in harsh conditions (flame retardance, and inertness to corrosive and different temperature environments), and excellent mechanical properties. The dip-coating strategy proposed for fabrication of the SMS is very simple and easy to scale up, since it does not use a complicated process or sophisticated equipment. Therefore, we believe that this sponge is a promising candidate for water remediation, the clean-up of large-area oil spills, and oil recovery.

## Experimental Methods

### Materials and synthesis

In this study, MoS_2_ bulk crystals (0.2 g, 99%, Alfa Aesar) were added to 200 ml of ethanol in a 250 ml capacity, flat-bottomed beaker. These samples were sonicated continuously for 24 h using a horn probe sonic tip. They were then centrifuged at 1000 rpm for 15 min to obtain a MoS_2_ nanosheets dispersed in ethanol solution. A piece of commercial melamine-formaldehyde sponge was first cleaned with acetone and distilled water successively using an ultrasonic cleaner, followed by drying in a vacuum oven at 100 °C for several hours to completely remove all moisture. The dried sponge was cut into smaller size (2 × 2 × 4 cm^3^) and was then dipped into a dispersion of MoS_2_ nanosheets in ethanol, and finally dried in a vacuum oven at 100 °C for 2 h. Different loadings of MoS_2_ nanosheets on the sponges were controlled by repeating the “dipping and drying” process.The density of the fabricated MoS_2_-based sponges was about 0.0101 g/cm^3^.

### Material characterization

SEM images were collected in a JSM-6610LV scanning electron microscope. TEM images were produced in JEOL JEM-2010 transmission electron microscope. Water contact angles were measured using a Rame-hart Model 250 Goniometer at room temperature, and the volume of distilled water droplets was 10 μL. The compression tests were performed on an Instron universal testing machine with a compressive rate of 20 mm/min.

## Additional Information

**How to cite this article**: Gao, X. *et al*. Flexible Superhydrophobic and Superoleophilic MoS_2_ Sponge for Highly Efficient Oil-Water Separation. *Sci. Rep.* **6**, 27207; doi: 10.1038/srep27207 (2016).

## Supplementary Material

Supplementary Information

Supplementary Movie 1

Supplementary Movie 2

Supplementary Movie 3

Supplementary Movie 4

Supplementary Movie 5
