# Supplementary material for: Flexible Superhydrophobic and Superoleophilic MoS2 Sponge for Highly Efficient Oil-Water Separation
Source: Sci Rep. 2016 Jun 2;6:27207. doi: 10.1038/srep27207 (PMC4897779; doi:10.1038/srep27207)
Supplement: Supplementary Information [file srep27207-s1.doc]

Supporting Information

**Flexible** **Superhydrophobic and Superoleophilic MoS_2_ Sponge for Highly Efficient** **Oil-Water Separation**

Xiaojia Gao^1^, Xiufeng Wang^1*^, Xiaoping Ouyang^1^, and Cuie Wen^2*^

^1^School of Materials Science and Engineering, Xiangtan University, Hunan411105, China

^2^School of Aerospace, Mechanical and Manufacturing Engineering, RMIT University, Bundoora, Victoria 3083, Australia

**Captions of Supporting Materials**

**Figures:**

**Figure S1**. Optical image of MoS_2_ nanosheets on glass slides by dip coating method.

**Figure S2**. **(a-c)** TEM image of MoS_2_ nanosheets, which are composed of multi-layer MoS_2_ based building blocks. **(d)** SAED patterns of MoS_2_ nanosheets.

**Figure S3**. Photograph of hydrophobic behavior of the MoS_2_ film on an aluminum substrate by dip coating method, the inset is optical image of a water droplet on MoS_2_ film at a WCA of 122°.

**Figure S4.** **(a)** The dyed water on the surface of the raw melamine-formaldehyde (MF) sponge. **(b)** Video snapshots of a drop of water absorbed by the raw MF sponge. **(c)** The diesel oil on the surface of the raw MF sponge. Inset: The oil contact angle of the raw sponge.

**Figure R5.** **(a)** Effect of diesel oil absorption-squeezing cycles on the WCA of the SMS. **(b)** The Water (dyed in red) droplets as quasi-spheres and diesel oil trace on the surface of the SMS after 20 cycles of the absorption-squeezing test.

**Figure S6.** Photographs of the oil collection apparatus continuously collecting floating gasoline on a moving water (dyed in red) surface

**Figure S7.** Optical photographs of the processes that the collection of diesel could be maintained for over 10 hrs (arrows indicating the direction of flow).

**Figure S8**. Snapshots of flame-retardant behavior of polymer sponge **(a)**, MoS_2_ sponge in air **(b)**, and combustion process of oil-saturated MoS_2_ sponge in air **(c)**.

**Figure S9**. Gravimetric absorption capacity and quality of MoS_2_ sponge as a function of combustion desorption cycles.

**Figure S10**. Mechanical properties of the MoS_2_ sponge: **(a)** Stress-strain curves of the MoS_2_ sponge with different set strain of 20, 40, 60 and 80%, respectively. The unloading curves almost return to the initial points, indicating the good flexibility. **(b)** Stress-strain curves of the MoS_2_ sponge with 50% set strain after 1 cycle and 1000 cycles.

**Figure S11**. Optical images of MoS_2_ sponges under **(a)** the bend and **(b)** twist states, showing their excellent mechanically flexibility.

**Figure S12**. The compression-recovery process of MoS_2_ sponge after one cycles of burning.

**Movies:**

**Movie 1**. Adsorption process of rapeseed oil (dyed with Sudan III) using MoS_2_ sponge (SMS);

**Movie 2.** Adsorption process of chloroform using SMS;

**Movie 3**. The oil-collection performance of SMS by using pump apparatus.

**Movie 4**. The oil-collection performance of SMS pumping apparatus on a water surface with simulated waves.

**Movie 5**. The oil-collection endurance of SMS pumping apparatus, which can be maintained for more than 10 hrs without an obvious decrease in flux.

**
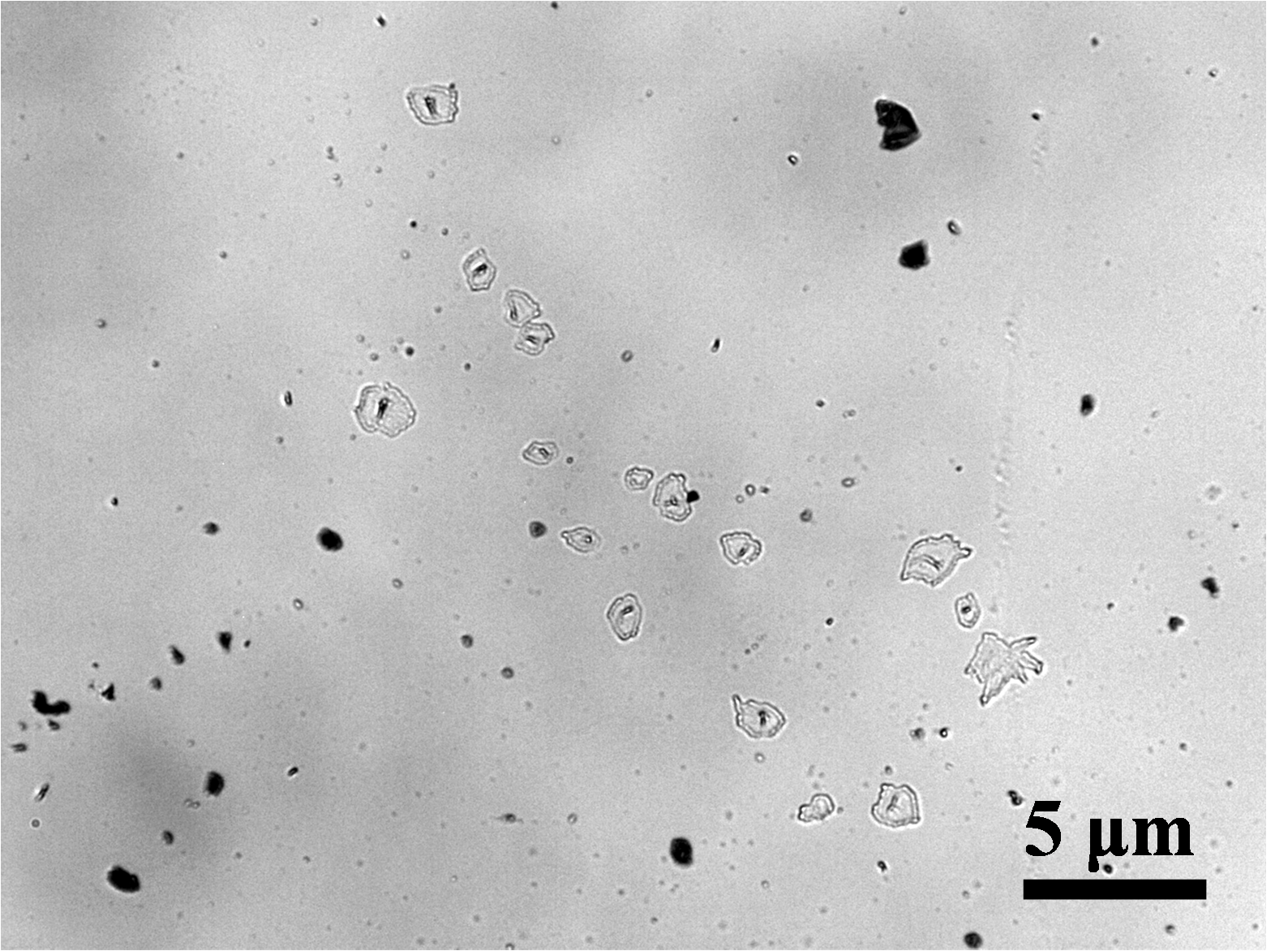
**

**Figure S1**.

**
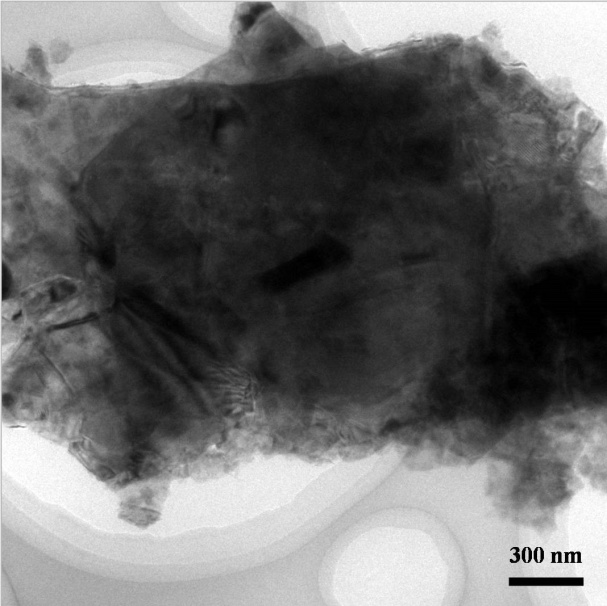

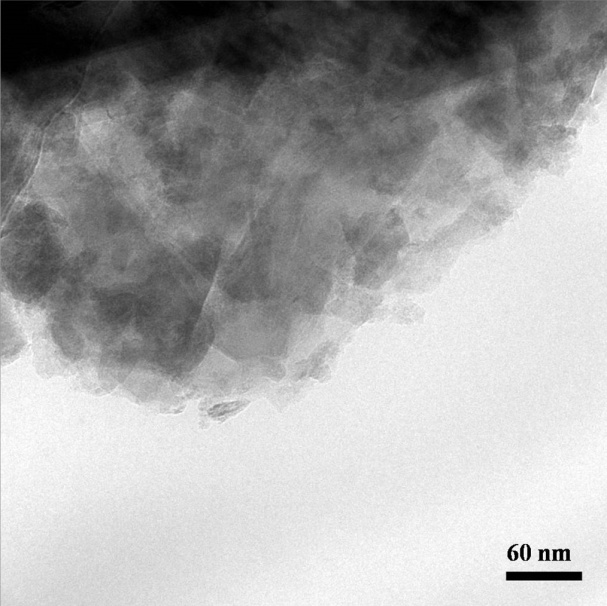
**

(d)

(c)

(b)

(a)


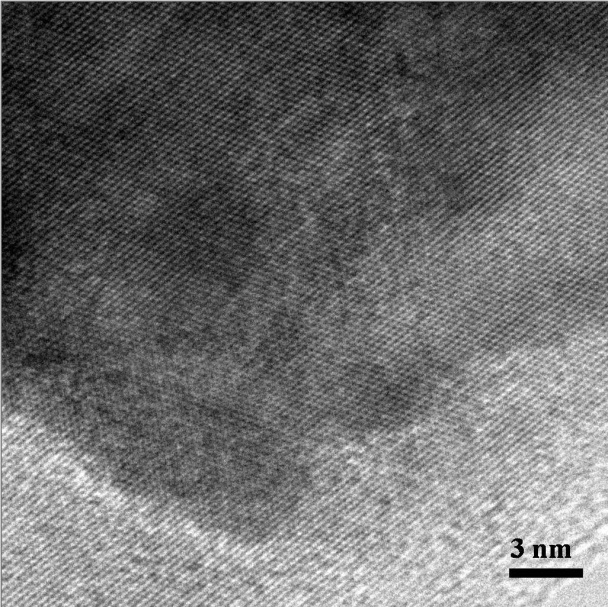

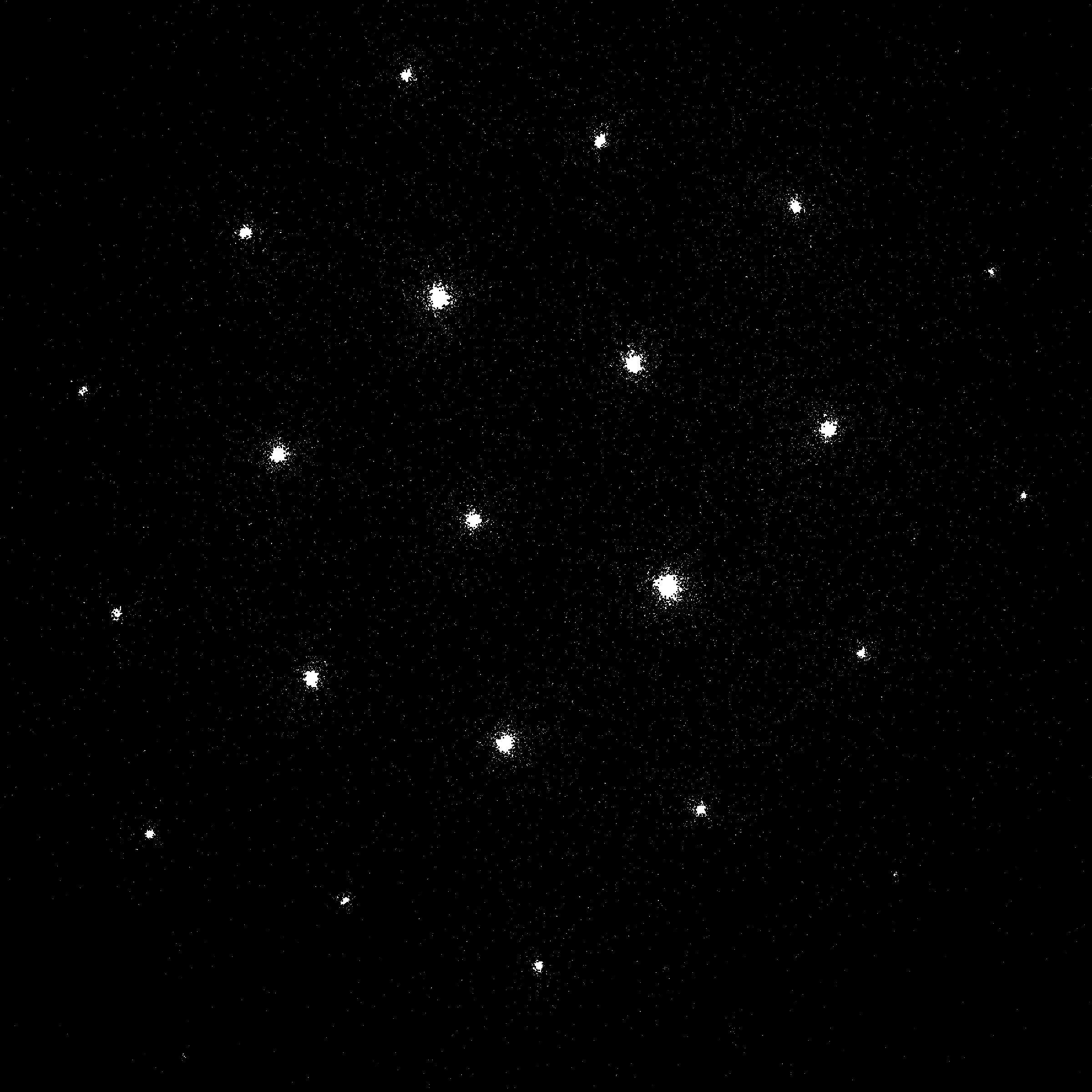


**Figure S2**.


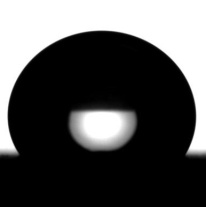

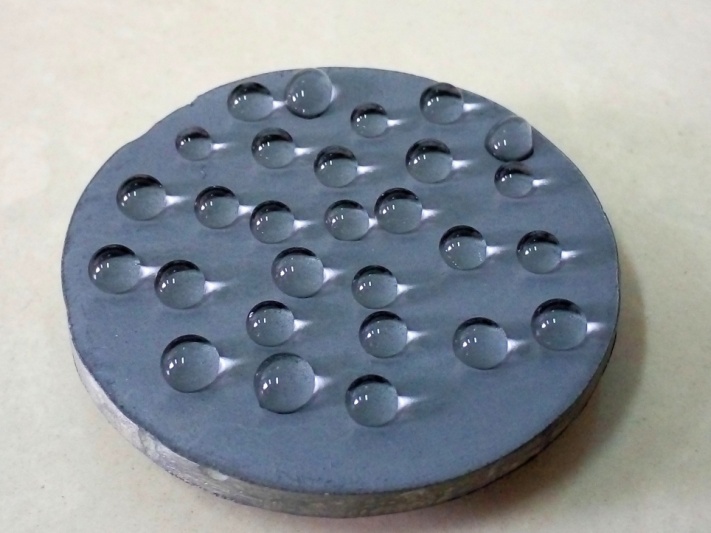


**10 mm**

**Figure S3**.


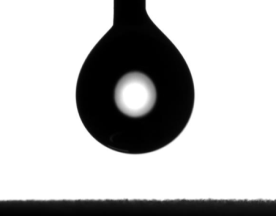

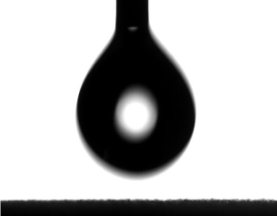

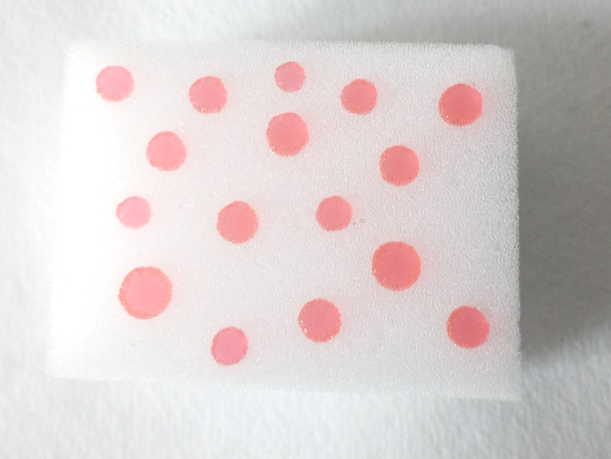


**0ms**

**33ms**

**(b)**

**(a)**


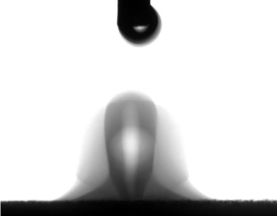

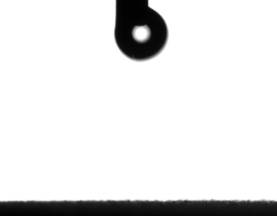
0

**99ms**

**66ms**


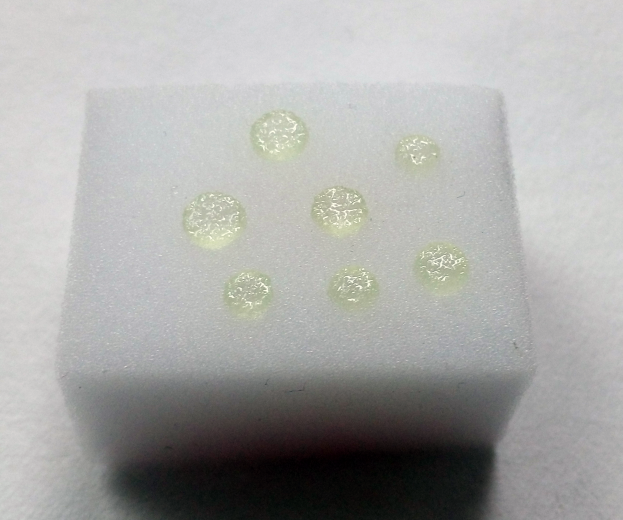


**(c)**


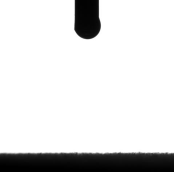


**Figure S4**.

(a)


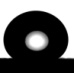

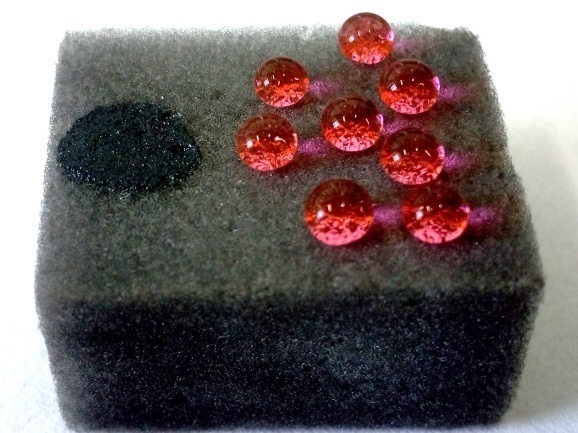


(b)

**Figure S5.**

**up**

**up**


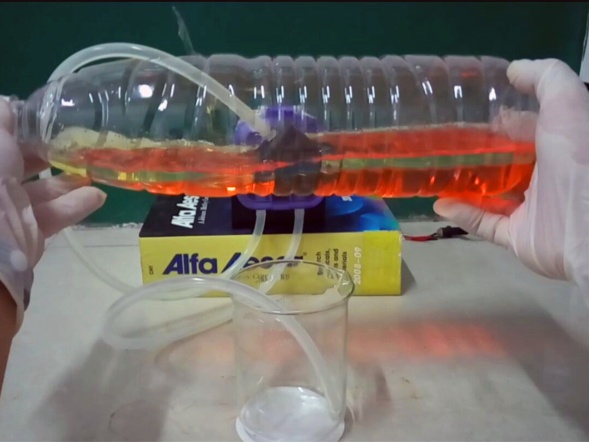

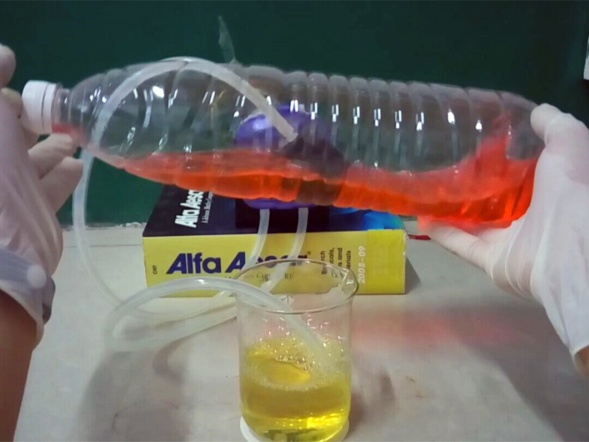


**down**

**down**


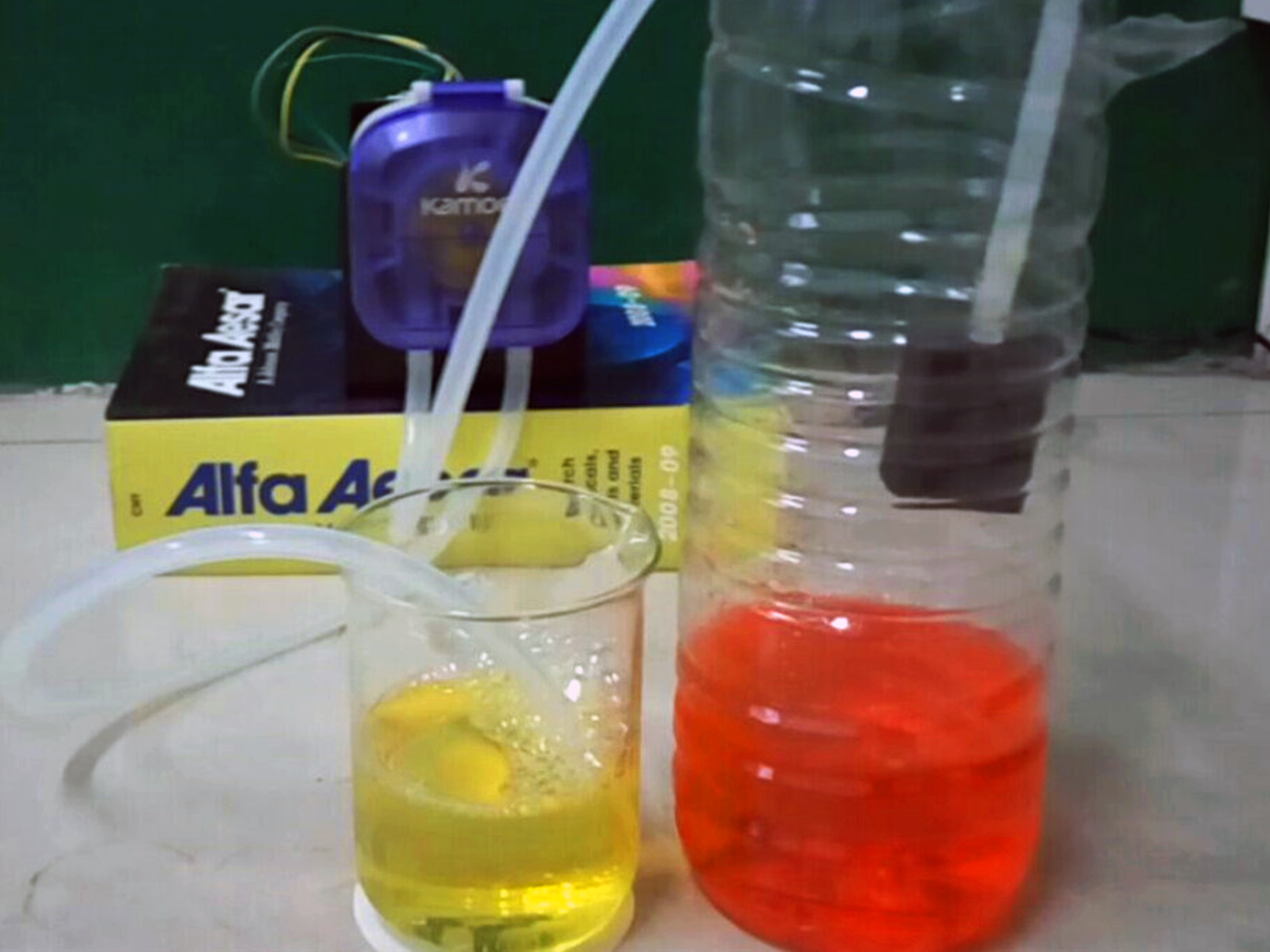


**oil**

**water**

**Figure S6.**


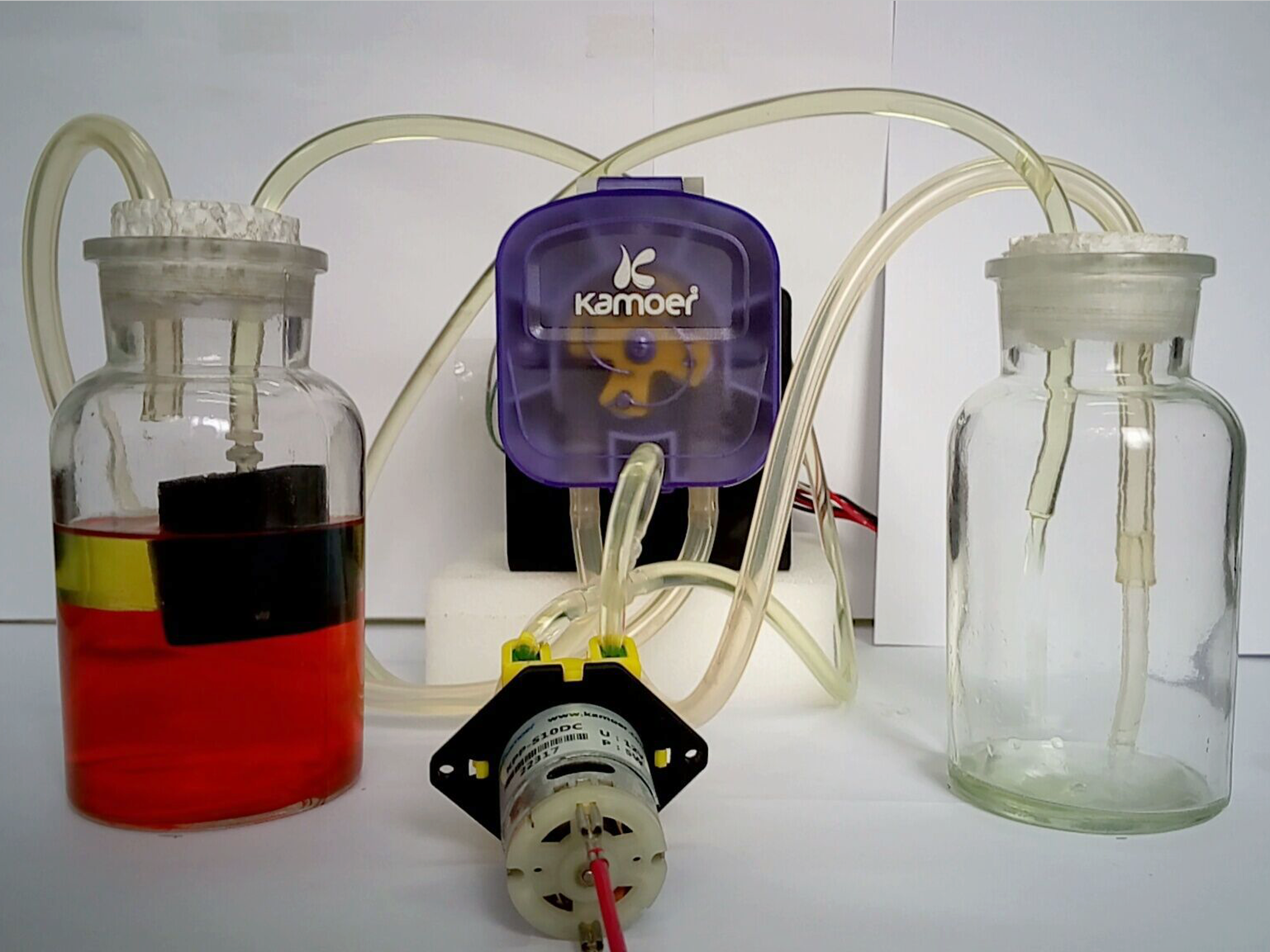

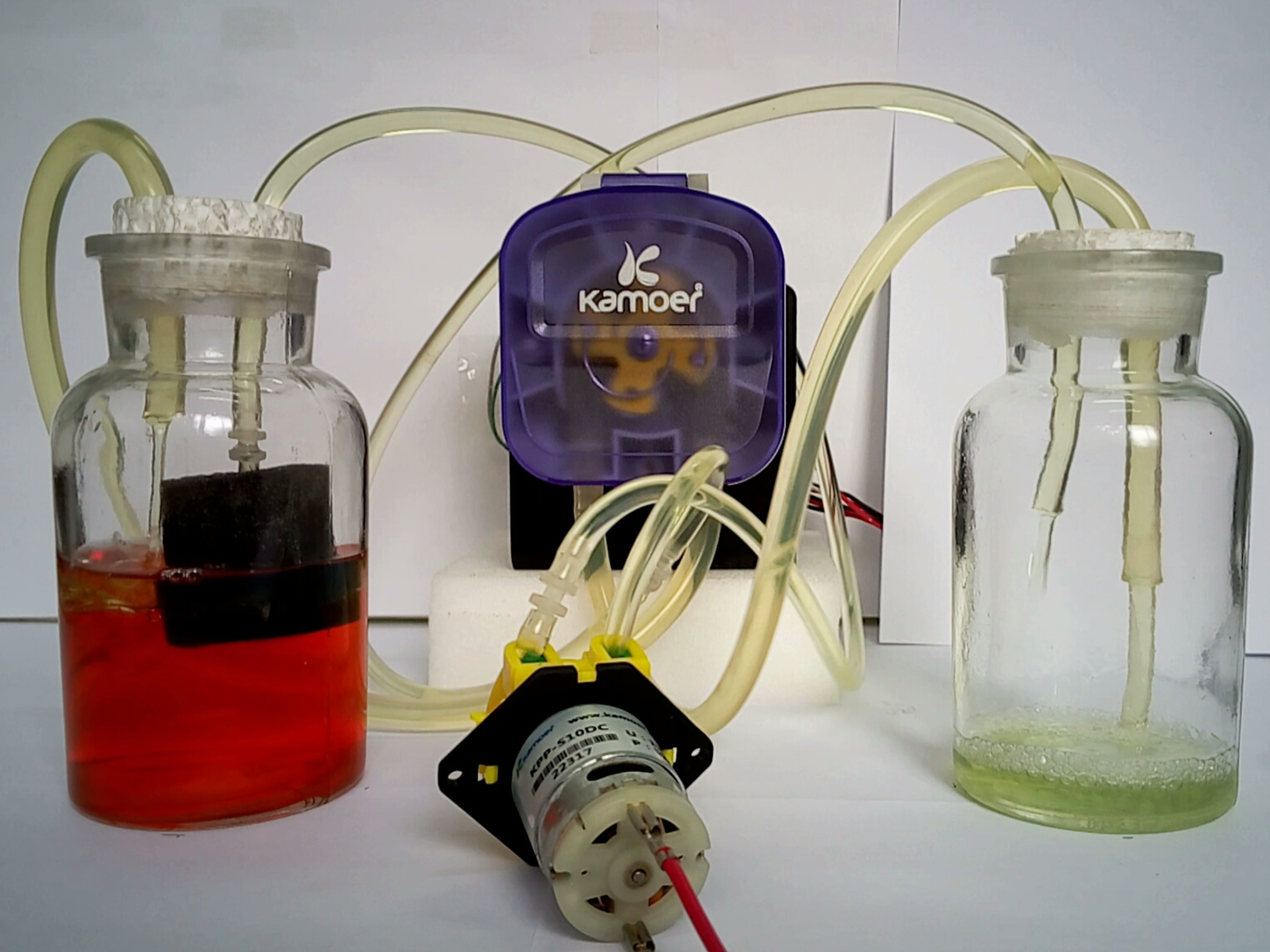


10 hrs later

Figure S7.


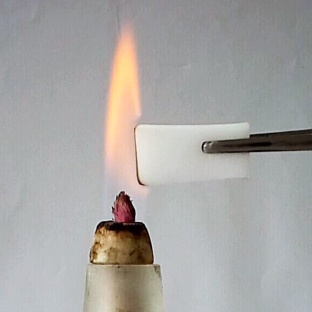

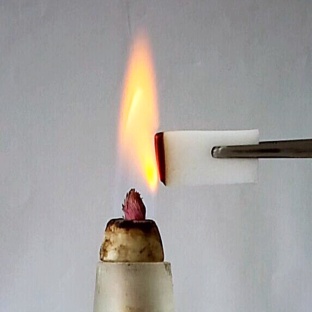

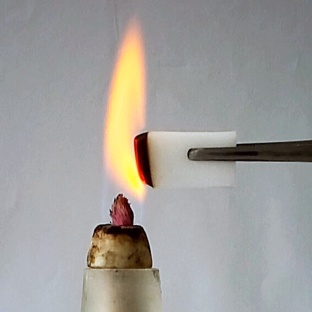

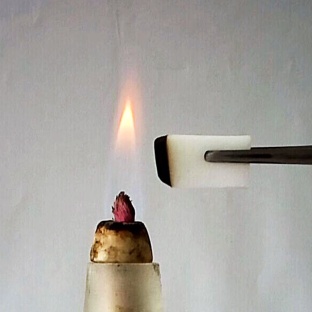


**0 s**

**5 s**

**11 s s**

**12 s**

**0 s**

**5 s**

**9 s**

**(a)**

**(b)**


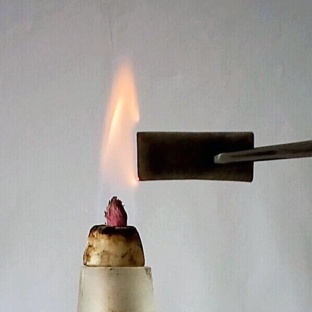

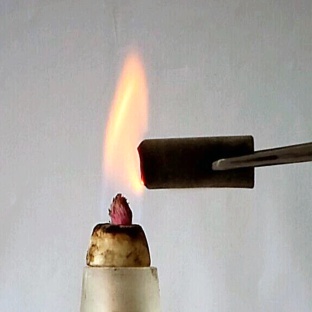

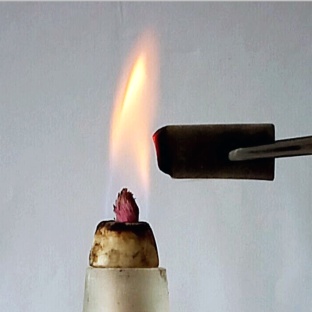

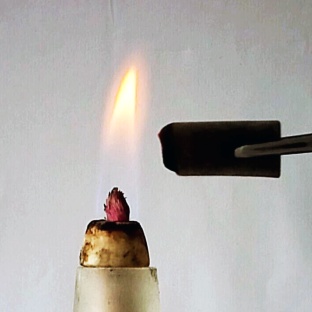


**10 s**

**(c)**


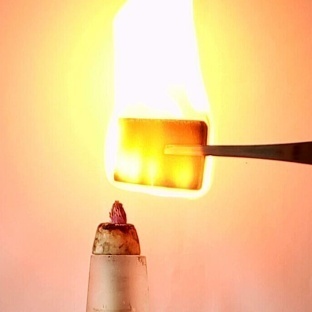

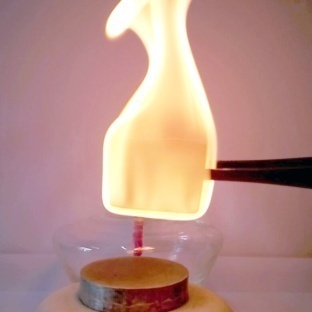

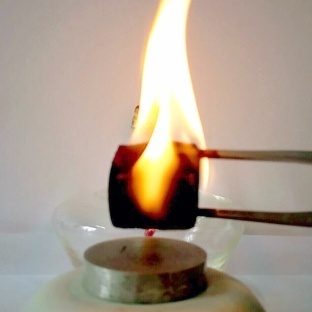

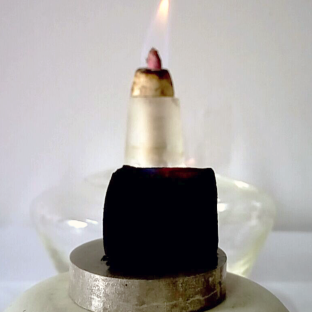


**50 s**

**2 s**

**0 s**

**70 s**

**Figure S8**.

**Figure S9**.

(a)

(b)

**Figure S10**.

**(a)**

**(b)**


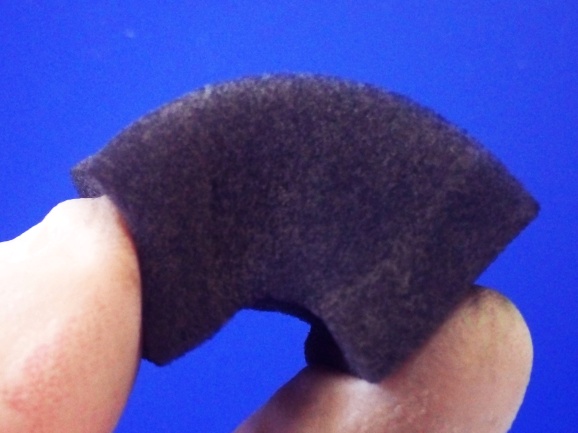

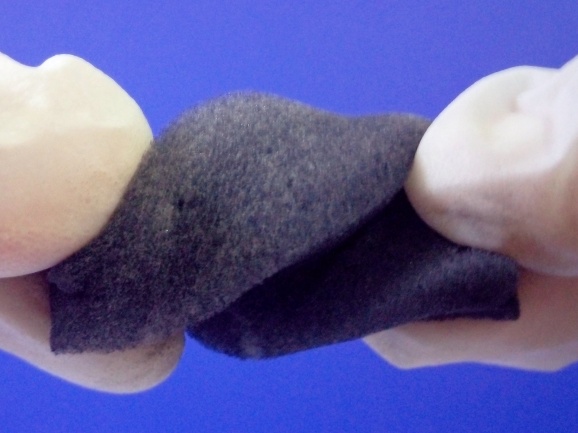


**Figure S11**.


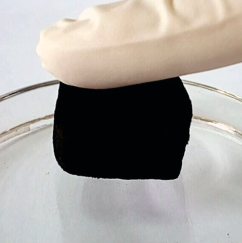

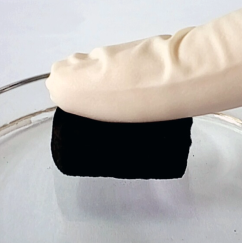

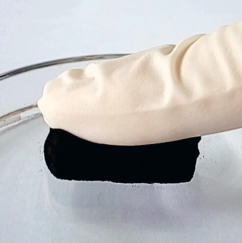

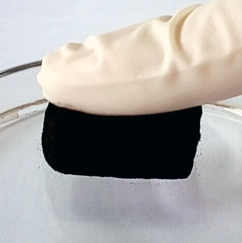

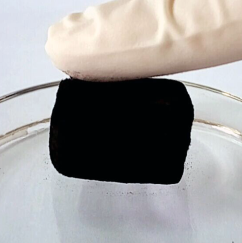


**Figure S12**.
